# Night-shift work and psychiatric treatment. A follow-up study among employees in Denmark

**DOI:** 10.5271/sjweh.4008

**Published:** 2022-03-31

**Authors:** Karen Albertsen, Harald Hannerz, Martin L Nielsen, Anne Helene Garde

**Affiliations:** 1 TeamArbejdsliv ApS, Valby, Denmark; 2 National Research Centre for the Working Environment, Copenhagen, Denmark; 3 Lægekonsulenten.dk, AS3 Companies, Viby J, Denmark; 4 Department of Public Health, University of Copenhagen, Copenhagen K, Denmark

**Keywords:** antidepressant, anxiolytics mental health, mood disorder, night work, occupational health, prescription drug, psychiatric hospital treatment, psychotropic medicine, shift work, stress-related disorder

## Abstract

**Objectives:**

We aimed to test the hypotheses that night-shift work is associated with an increased incidence of (i) redeemed prescriptions for psychotropic medicine and (ii) psychiatric hospital treatment due to mood, anxiety or stress-related disease. Moreover, we aimed to assess whether (iii) the effect of night-shift work on the rates of antidepressants differs from the effects on the rates of anxiolytics and (iv) the association between night-shift work and psychotropic medicine is affected by long working hours.

**Methods:**

Full-time employees who participated in the Danish Labor Force Survey sometime in the period 2000–2013 (N=131 321) were followed for up to five years in national registers for redeemed prescriptions and psychiatric hospital treatment. The analyses were controlled for sex, age, weekly working hours, calendar time of the interview and socioeconomic status.

**Results:**

We detected 15 826 cases of psychotropic drug use in 521 976 person-years at risk and 1480 cases of hospitalization in 636 673 person-years at risk. The rate ratio (RR) for psychotropic drugs was estimated to be 1.09 [99% confidence interval (CI) 1.02–1.16] for night-shift versus no night-shift work. The corresponding RR for psychiatric hospital treatment was 1.11 (95% CI 0.95–1.29). The odds of redeeming a prescription for antidepressants rather than anxiolytics was independent of night-shift work: 1.09 (95% CI 0.96–1.24), and we found no interaction effect between night-shift work and working hours (P=0.26).

**Conclusion:**

As it appears in the general working population in Denmark, night-shift work is not an important predictor of mental ill health.

Shift work is common in many industries, eg, within manufacturing, transportation and healthcare, and therefore possible negative health outcomes will potentially affect many workers. It is estimated that around 20% of the working population in Europe are working in shifts and work the night shift at least once per month (1). Health outcomes associated with shift work have been studied intensively, and shift work has been associated with a range of negative health consequences (2). Also, the possible link between working in shifts and decreased mental health has been examined in a range of studies (3–5).

Biological as well as social and environmental factors may interact as underlying mechanisms behind an association between shift work and mental health. Sleep disturbances, circadian misalignment (6), and abnormal stress responses (7) may affect mood, and vigilance (8) and in turn the regulation of emotions (7). Working in shifts may impact the work–life balance (8), marital satisfaction (10), and social life (11) negatively. Furthermore, increased risk for exposure to traumatic events is prevalent in some of the sectors where shift work is common, eg, healthcare and protective services (12, 13). Traumatic events are to some degree inherent to the work demands within these sectors, and not caused by shift work, but often associated with – and to some degree not possible to disentangle totally from – the effect of shift work.

The association between shift work and mental health issues is well documented in cross-sectional studies. A recent review based on 21 cross-sectional and 4 prospective studies found shift work to be associated with increases in depressive symptoms (12 studies), anxiety (2 studies), and depression and anxiety (7 studies) (4). A meta-analysis conducted on 9 cross-sectional and 2 prospective studies also concluded that night-shift work was significantly associated with an increased risk of depression (14).

All though, shift work has been associated with the development of mental health issues also in prospective studies (15), other studies have not prospectively found any association between shift work and mental ill health (16–20).

A recent meta-analysis included data from seven longitudinal studies (covering 28 431 unique participants) and found shift work to be associated with increased overall risk of adverse mental health outcomes and particularly with depression (3). They also found that the heterogeneity in effects among the studies was substantial, and mainly due to sex differences, with higher risk among female than among male shift workers. The authors mentioned that the difference may be partly explained by the higher risk of depression among women than men in the general population. Further they mentioned the lack of occupational information in most studies as an important limitation to study potential moderating effects.

Thus, despite many studies, and a couple of reviews and meta-analyses in the field, it is still essential to investigate possible effects of shift work on mental health issues in large, prospective studies in order to obtain more clarity on potential effect sizes among men and women and to pay attention to potential differential effects eg, according to different diagnoses, occupational groups and nations. In a previous study (20) (not included in any of the previously mentioned meta-studies), we estimated the prospective associations between shift work and use of psychotropics in a large (N=19 259) Danish sample and found the rate ratio for incidence of redeemed prescriptions for psychotropic drugs among shift workers to be 1.09 [95% confidence interval (CI) 0.99–1.21] (20, 21).

Results from our secondary (hypothesis-generating) analyses in the same study suggested that excessive overtime work (>48 hours/week) may be an important risk factor for mental disorders among shift workers [rate ratio (RR) 1.51, 95% CI 1.15–1.98], although studies do not suggest overtime work as a risk factor for mental disorder in the general working population in Denmark (21–23, 34). Further, results from the secondary analyses suggested increased incidence of use of hypnotics, sedatives and antidepressants and decreased incidence of use of anxiolytics among shift workers (20). To our knowledge, the results of the abovementioned secondary analyses have not been observed in any other study.

Based on these secondary analyses (20), we generated four different hypotheses:

1. Overtime work (> 48 hours/week) increases the risk of mental health problems among shift workers.
2. Results from a previous study showed increased use of hypnotics, sedatives and antidepressants among shift workers, suggesting that shift work increases the incidence of sleeping problems and depression while at the same time results showed decreased incidence of use of anxiolytics among shift workers, suggesting that shift work decreases the incidence of anxiety. These oppositely directed effects cancel out each other and give a non-significant total effect. According to this interpretation, different incidences of diagnoses among shift workers versus others should be hypothesized.
3. Anxiety may be treated with anxiolytics as well as with antidepressants. Since workers on night shifts needs to stay awake and one of the side-effects of anxiolytics may be drowsiness, there may be an increased likelihood that a night-shift worker with anxiety will be prescribed antidepressants rather than anxiolytics. Diagnosis associated with hospital treatment are based on the clinical picture and not side-effects of drugs, so this hypothesis will get support if the odds ratio (OR) for antidepressants versus anxiolytics is increased while the OR for hospital treatment due to depression versus anxiety among shift workers versus non-shift workers is not.
4. The increased incidence of use of hypnotics, sedatives and antidepressants and decreased incidence of use of anxiolytics among shift workers that we found in our previous study (20) may be a coincidence.

The present study aimed at testing the hypotheses that night-shift work is associated with an increased incidence of redeemed prescriptions for psychotropic drugs, and psychiatric hospital treatment due to mood, anxiety or stress-related disease.

Another aim was to test the hypotheses that were generated from our previous study in a dataset that is independent of and larger than those previously used.Firstly, we investigated the prospective association between night-shift work and incident use of psychotropic medicine and tested for effects of interactions between night-shift work and age, sex and socio-economic status (SES), respectively. Secondly, we investigated whether excessive overtime work was an important risk factor among night-shift workers. Thirdly, we tested differential prospective effects on anxiolytics versus hypnotics, sedatives, and antidepressants, and on psychiatric hospital treatment for mood versus anxiety and stress-related disorders.

## Method

We include here a brief description of the material and methods of the study. A detailed description can be found in the study protocol (22), which was peer-reviewed and published before we conducted the analysis. The protocol defines two major studies, one of them focuses on effects of night-shift work (results reported here), the other focuses on effects of long working hours (23).

### The data material

Individual participant data on night-shift work were retrieved from the Danish Labor Force Survey (DLFS) (24). The DLFS data were linked to person-based data from national registers, which cover the entire population of Denmark. Data from the Central Person Registry (25) were used for the linking of data. The National Prescription Registry (26) provided data on prescriptions of psychotropic medicine. The Psychiatric Central Research Register (27) provided data on psychiatric hospital treatments, and the Employment Classification Module (28) on industry, socioeconomic status, migrations, and deaths. The DLFS is based on random samples drawn each quarter of each calendar year since 1994. Data were collected by means of telephone interviews during the time-period spanned by the present study. Participants were 15–74-year old inhabitants of Denmark and invited to be interviewed four times during a period of approximately 1.5 years. The response rates have decreased from 70% in 2002 to 53% in 2013. The primary analyses of the present study are based on the participants’ first interview in the calendar period 2000–2013.

### Inclusion criteria

**Figure 1:**
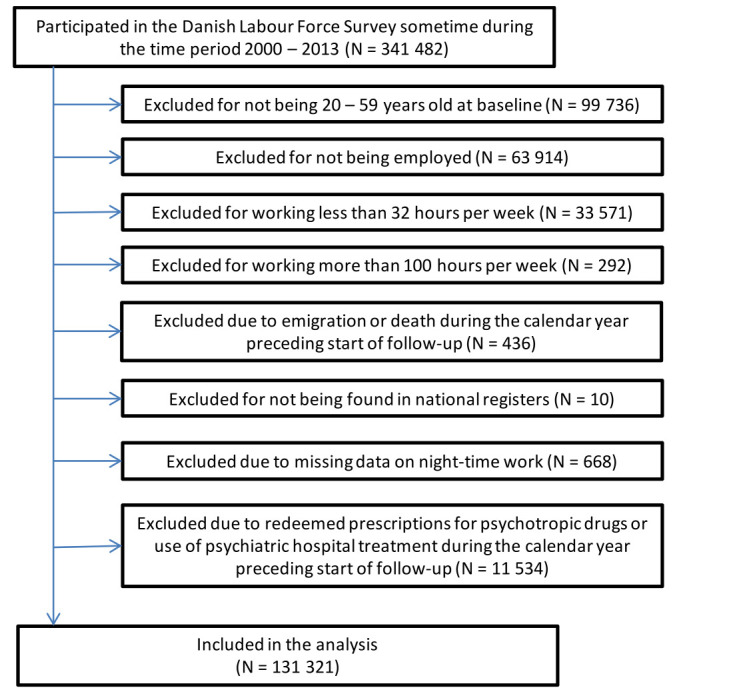
Flowchart of participants.

The study included people who: were 20–59 years old at the start of the follow-up period; responded to DLFS sometime during the calendar years 2000–2013; were employed with 32–100 usual working hours a week at the time of the interview; did not redeem a prescription for any type of psychotropic drug (ATC: N05-N06) and did not receive hospital treatment for a primary diagnosis of any type of mental disorder (ICD-10: F00-F99) during a one-year period preceding the start of the follow-up. As they reported >100 working hours per week, 297 persons were excluded. Only 1.2% of the included participants worked >60 hours a week. Figure 1 presents a flowchart over the exclusion and inclusion of participants.

### Clinical endpoints

The following outcomes were regarded each at a time (i): redeemed prescriptions for any type of psychotropic medicine, ie, drugs in the ATC-code category N05 (psycholeptica) or N06 (psychoanaleptica); and (ii) psychiatric hospital treatment with a mood, anxiety or stress-related disorder (ICD-10: F30 – F41 or F43) as principal diagnosis.

### Night-shift work

Participants who responded either “yes, regularly” or “yes, occasionally” to the question about night-shift work at baseline were defined as being exposed. Those who responded “no…” were defined as being unexposed to nighttime work. Before 2001, the participants were simply asked whether they worked at night, but from 2001 onward, the question has been whether they worked at night during the last four weeks. Until 2006 the response categories were “yes, regularly,” “yes, occasionally,” and “no, never”. From 2007 and onward the response categories were expanded to “yes, regularly” (ie, more than half of the working days in the last four weeks), “yes occasionally” (ie, at least once within the last four weeks, but less than half of the working days), and “no, not within the last four weeks.”…. The exposure categories were based on the status at baseline; changes in exposure status over time were not taken into consideration in the primary analyses.

### Follow-up

The follow-up started at the end of the same year as the baseline interview with the participant was conducted. Follow-up ended when one of the following events occurred: the subject reached the clinical endpoint of the analysis; the subject emigrated; the subject died; five years had passed since start of follow-up; or the study period ended (31 December 2014 for psychotropic medicine; 31 December 2017 for psychiatric hospital treatment). Hence, the participants were followed for a maximal period of five years. The reason for stopping at five years was that a too long follow-up period would dilute the effect, since night workers may become day workers and vice versa during the follow-up.

### Statistical analyses

Poisson regression was used to estimate rates of redeemed prescriptions for psychotropic medicine and psychiatric hospital treatment due to mood, anxiety, or stress-related disorders, separately, as a function of night-shift work (yes versus no). The analyses were controlled for weekly working hours (32–40; 41–48; >48 hours/week), sex, age (10-year classes), calendar time of the interview (2000–2004; 2005–2009; 2010–2013) and SES (legislators, senior officials and managers; professionals; technicians and associate professionals; workers in occupations that require skills at a basic level; workers in elementary occupations; and gainfully occupied people with an unknown occupation).

The logarithm of person years at risk was used as offset. Likelihood ratios were used to test for statistical significance. For redeemed prescriptions of psychotropic medicine, we tested the following effects, each at the significance level 0.01: (i) main effect of night-shift work; (ii) effect of interaction between age and night-shift work; (iii) effect of interaction between sex and night-shift work; (iv) effect of interaction between SES and night-shift work; and (v) effect of interaction between weekly working hours and night-shift work. In order to avoid publication bias in future meta-analyses, we have chosen to present results of all stratified analyses, even though the interaction effects were not significant. If eg, gender differences were only presented if the difference were statistically significant, this would lead to over-estimation in potential meta-analyses.

For psychiatric hospital treatment due to mood, anxiety or stress-related disorders, we tested for a main effect of night-shift work at the significance level 0.05. As the incidence of hospital treatment is substantially lower than the incidence of medical treatment, we did not have the statistical power necessary to test for interaction effects. We chose a significance level at 0.01 for the analyses of psychotropic medicine to correct for multiple testing. For the analysis of hospitalizations, we only tested one hypothesis and could therefore use a significance level of 0.05.

A sensitivity analysis was conducted to find out if the estimated strength of the association between night-shift work and redeemed prescriptions for psychotropic drugs would increase when exposure is more stable over time. In this analysis, we only included employees who were employed ≥32 working hours a week according to their first as well as their last interview and belonged to the same category in relation to night-shift work (yes versus no) during their last interview as they did during their first interview (over a period of 1.5 year, see data material). In another sensitivity analysis, we estimated relapse rate ratios among employees with a past record of psychiatric treatment. Sensitivity analyses were, moreover, conducted to compare results obtained (i) with and without exclusion of former and current cases of psychiatric treatment, and (ii) with and without control for industrial sector. The methods and results of the sensitivity analyses are given in the supplementary material (www.sjweh.fi/article/4008).

In the sensitivity analyses, we estimated a series of RR and presented these with 99% CI. It should be noted that we do not regard the sensitivity analyses and their CI as statistical significance tests. They may, however, strengthen, weaken, or invalidate statistical conclusions of the primary analyses.

## Results

The criteria for inclusion in the primary analyses were fulfilled by 131 321 participants (figure 1). Among the included, we observed a total of 15 826 cases of redeemed prescriptions for psychotropic drugs in 521 976 person years at risk and 1480 cases of psychiatric hospital treatment due to mood, anxiety or stress-related disease in 636 673 person years at risk. Of the hospital treatment cases, 22% were inpatients, 53% were outpatients and 25% were emergency ward patients. The diagnoses among the cases were distributed as follows: F30 manic episode 0.5%; F31 bipolar affective disorder 2.4%; F32 depressive episode 25.8%; F33 recurrent depressive disorder 10.2%; F34, F38, F39 persistent, other or unspecified affective mood disorders 0.5%; F40 phobic anxiety disorders 3.0%; F41 other anxiety disorders 8.6%; F43 reaction to severe stress, and adjustment disorders 48.9%.

In the main analyses, we found that night-shift work was statistically significantly associated with redeemed prescriptions for psychotropic drugs (P=0.0007), with RR estimated at 1.09 (99% CI 1.02–1.16) (see table 1). We did not find any increased incidence in the group working very long hours (>48 hours/week), but we did find indication of an increased risk among those working in night-shifts and 41–48 hours/week; RR 1.20 (99% CI 1.00–1.44).

**Table 1:** Rate ratio (RR) with 99% confidence interval (CI) for incident use of psychotropic drugs, as a function of night-shift work among employees in Denmark 2000–2013.

Type of population	Night-shift work	Persons	Person years	Cases	Crude RR	RR	99% CI
All employees^a^	Yes	16 651	65 557	2026	1.02	1.09	1.02–1.16
	No	114 670	456 418	13 800	1.00	1.00	-
Male employees^b^	Yes	11 118	44 390	1167	1.09	1.06	0.98–1.16
	No	59 861	240 875	5796	1.00	1.00	-
Female employees^b^	Yes	5533	21 167	859	1.09	1.11	1.01–1.21
	No	54 809	215 543	8004	1.00	1.00	-
Employees with >48 working hours a week^c^	Yes	2356	9514	290	1.03	1.03	0.86–1.23
	No	5637	23 204	688	1.00	1.00	-
Employees with 41–48 working hours a week^c^	Yes	1964	8211	247	1.11	1.20	1.00–1.44
	No	11 628	48 953	1321	1.00	1.00	-
Employees with 32–40 working hours a week^c^	Yes	12 331	47 833	1489	1.01	1.08	1.00–1.16
	No	97 405	384 261	11791	1.00	1.00	-
20–29 years^d^	Yes	2822	11 089	285	1.10	1.14	0.97–1.35
	No	19 307	76 986	1803	1.00	1.00	-
30–39 years^d^	Yes	4828	19 345	562	1.02	1.08	0.96–1.22
	No	30 797	125 046	3549	1.00	1.00	-
40–49 years^d^	Yes	5059	19 903	639	1.00	1.06	0.95–1.19
	No	33 043	129 986	4164	1.00	1.00	-
50–59 years^d^	Yes	3942	15 220	540	1.03	1.09	0.96–1.22
	No	31 523	124 400	4284	1.00	1.00	-
Legislators, senior officials, and managers^e^	Yes	438	1608	43	1.01	1.08	0.71–1.63
	No	4184	16 117	426	1.00	1.00	-
Professionals^e^	Yes	2963	10 289	309	1.15	1.19	1.02–1.39
	No	22 525	83 094	2179	1.00	1.00	-
Technicians and associate professionals^e^	Yes	2693	11 508	352	1.04	1.04	0.90–1.20
	No	20 738	86 284	2541	1.00	1.00	-
Workers in occupations that require skills at a basic level	Yes	6776	27 112	822	0.98	1.07	0.97–1.18
	No	45 734	184 270	5722	1.00	1.00	-
Workers in elementary occupations^e^	Yes	1384	5504	204	1.03	1.14	0.94–1.39
	No	9327	37 590	1351	1.00	1.00	-
Gainfully occupied people with an unknown occupation^e^	Yes	2397	9536	296	0.96	1.05	0.90–1.24
	No	12 162	49 063	1581	1.00	1.00	-

a Adjusted for sex, age, weekly working hours, calendar time of the interview and socioeconomic status

b Adjusted for age, weekly working hours, calendar time of the interview and socioeconomic status

c Adjusted for sex, age, calendar time of the interview and socioeconomic status

d Adjusted for sex, weekly working hours, calendar time of the interview and socioeconomic status

e Adjusted for sex, age, weekly working hours and calendar time of the interview

The association between night-shift work and psychiatric hospital treatment due to mood, anxiety or stress-related disease was estimated at RR 1.11 (95% CI 0.95–1.29) (table 2).

**Table 2:** Rate ratio (RR) with 95% confidence interval (CI) for psychiatric hospital treatment due to mood, anxiety or stress-related disorders, as a function of night-shift work among employees in Denmark in the calendar years 2000–2013

Night-shift work	Persons	Person years	Cases	Crude RR	RR	95% CI
Yes	16 651	80 288	202	1.10	1.11	0.95 - 1.29
No	114 670	556 385	1278	1.00	1.00	-

* Adjusted for sex, age, weekly working hours, calendar time of the interview and socioeconomic status

Secondly, we tested interaction effects on redeemed prescriptions for psychotropic drugs and did not find any statistically significant interaction between night-shift work and age (P=0.83), sex (P=0.48), SES (P=0.61), or weekly working hours (P=0.26).

Compared to non-shift workers, night-shift workers did not have higher odds to receive antidepressants rather than anxiolytics [OR 1.09 (95% CI 0.96-1.24)] (table 3). The same holds for the odds of receiving hospital treatment due to mood versus anxiety and stress-related disorders [OR 0.93 (95% CI 0.68–1.26)] (table 4).

**Table 3:** Odds ratio (OR) for antidepressants (AD) vs. anxiolytics (ANX) among employees in Denmark who redeemed a prescription for either AD or ANX sometime during the follow-up for psychotropic drugs, as a function of night-shift work at baseline. [CI=confidence interval.]

Night-shift work	AD cases	ANX cases	Odds of AD vs. ANX	Yes versus no to night-shift		
			
	N	N	OR	Crude OR	Adjusted OR^a^	95% CI
Yes	853	398	2.14	1.16	1.09	0.96–1.24
No	5703	3078	1.85	1.00	1.00	-

a Adjusted for sex, age, weekly working hours, calendar time of the interview and socioeconomic status.

**Table 4:** Odds ratio (OR) for mood (MD) vs. anxiety and stress-related disorders (ASD) among employees in Denmark who received psychiatric hospital treatment for mood, anxiety or stress-related disorders sometime during the follow-up period, as a function of night-shift work at baseline. [CI=confidence interval.]

Night-shift work	MD cases	ASD cases	Odds of MD vs. ASD	Yes versus no to night-shift		
			
	N	N	OR	Crude OR	Adjusted OR^a^	95% CI
Yes	82	122	0.67	1.01	0.93	0.68–1.26
No	515	771	0.67	1.00	1.00	-

a Adjusted for sex, age, weekly working hours, calendar time of the interview and socioeconomic status.

### Supplementary analyses

Results from all supplementary analyses can be found in the supplementary material.

*Amount of exposure*. In the first supplementary analysis (table S1), we studied whether the strength of the association between night-shift work and redeemed prescriptions for psychotropic drugs increased when the exposure was more stable over time. Result showed an increased risk among night-shift workers with stable exposure from first to last interview, slightly higher than the risk found in the main analyses [RR 1.13 (99% CI 1.02–1.25)].

A further supplementary analysis (see table S2) showed a higher risk for redeemed prescriptions of psychotropic drugs among employees with regular night-shift work (RR 1.14, 99% CI 1.05–1.24) than among employees with occasional night-shift work (RR 1.03, 99% CI 0.94–1.12).

Taken together, the supplementary analyses thus supported that more stable and more frequent exposure to night shift work may increase the risk for a redeemed prescriptions for psychotropic drugs.

There are relatively large groups of night-shift workers in nursing homes, home care etc. in Denmark, whose standard full-time work schedules imply an average of only 28 working hours a week. We therefore also tried to redefine the inclusion criterion from ≥32 hours a week to ≥ 28 hours a week and redefined the reference group to 28–40 hours a week. The statistical model was otherwise the same as in the primary analysis. The estimate decreased marginally after inclusion of this group, from 1.09 (table 1) to 1.08 (table S3).

*Relapse rates among employees with a past record of psychiatric treatment*. Among participants who received psychiatric treatment within the second to fifth but not within the first year prior to the start of follow up, we estimated the relapse RR for prescribed psychotropic drugs between employees with versus without night-shift work to be 0.97 (99% CI 0.83– 1.13) (Table S4).

*With and without exclusion of former and current cases of psychiatric treatment*. In the primary analysis, we excluded participants who received psychiatric hospital treatment or redeemed a prescription for psychotropic drugs during the calendar year preceding the start of the follow-up period. It is, however, possible that the results were influenced by cases that occurred earlier than one year prior to baseline. To explore this possibility, we conducted a sensitivity analysis in which we excluded all participants who received psychiatric treatment at any time within a five-year period prior to the start of follow-up. The results showed similar estimates in the cohort with exclusion of all previous cases as in the cohort used in the main analyses, and they showed no increased risk in the cohort of previously excluded cases (table S4).

Further, we repeated the main analysis without exclusion of prevalent cases, resulting in a marginally decreased estimate.

*Controlling for industrial sector*. We wanted to know if the results of the present study would change if we added industrial sector to the existing model, including the occupational-based SES. We conducted a sensitivity analysis, firstly controlling for (table S5) and secondly stratified by industrial sector (table S6 and Table 5). After control for industrial sector, the overall estimate decreased slightly and became insignificant (RR 1.06, 99% CI 0.99–1.13). The results of the industry-stratified analysis are given in table 5. As seen in the table, the confidence interval of each industry overlaps the confidence intervals of every other industry.

**Table 5:** Industry specific rate ratio (RR) with 99% confidence interval (CI) for incident use of psychotropic drugs, as a function of night-shift work among employees in Denmark in the calendar years 2000–2013.

Industry	Night work at baseline	Persons	Person years	Cases	RR^a^	99% CI
Agriculture, forestry, hunting and fishing	Yes	299	1169	29	1.11	0.66–1.86
	No	1813	7486	184	1.00	-
Manufacturing, mining and quarrying	Yes	3222	13 142	388	1.07	0.93–1.23
	No	19 558	80 311	2349	1.00	-
Construction	Yes	486	1941	52	1.13	0.78–1.64
	No	8343	34 359	820	1.00	-
Wholesale and retail trade; repair of motor vehicles	Yes	976	3898	104	1.09	0.84–1.41
	No	15 967	63 836	1708	1.00	-
Transporting and storage	Yes	2250	9063	266	1.07	0.88–1.29
	No	5492	22 513	653	1.00	-
Accommodation and food service activities	Yes	411	1588	46	0.96	0.63–1.46
	No	1636	6266	212	1.00	-
Human health and social work activities	Yes	3927	14 947	543	1.01	0.89–1.14
	No	18 558	72 126	2791	1.00	-
Other	Yes	4823	18 890	565	1.12	1.00–1.26
	No	41 814	164 021	4796	1.00	-
Missing	Yes	257	919	33	0.73	0.46–1.18
	No	1489	5502	287	1.00	-

* Adjusted for sex, age, weekly working hours, calendar time of the interview and socioeconomic status

## Discussion

In the primary analyses, we found night-shift workers to have a small but statistically significant, increased risk for mental health issues causing a redeemed prescription of psychotropic drugs (all types combined). Results from previous, prospective studies of shift work and mental health issues have used different outcome measures and reported estimates of different sizes. Bildt & Michelsen (15) found the odds for sub-clinical depression over a 5-year period to be 2.4 (95% CI 1.0–5.8) among females working in shift versus not working in shifts and 2.9 (95% CI 1.2–7.2) among men working in shifts. Other prospective studies have not found any significantly increased risk for mental health issues among shift workers (17–19). Driesen et al (17) reported, over a 10-years period the hazard ratio for the development of incident depressed mood among male shift workers to be 0.86 (95% CI 0.69–1.08) after adjustment for psychosocial work-related factors. Suwazano et al (18) found the odds for self-perceived mental conditions among men working in shifts versus not working in shifts to be 0.91 (95% CI 0.74–1.11) and among women to be OR 0.98 (95% CI 0.82– 1.18); and Lasalle et al (19) found in a mixed sample the odds for psychotropic drug use to be 1.05 (95% CI 0.80–1.39) among shift workers versus non shift workers (19). In our own previous study, we found the RR for prescription of psychotropic drugs among shift workers versus non shift workers to be of same size as in the present study but with a larger CI: 1.09 (95% CI 0.99–1.21). As it appears from the wide CI, the statistical power of most of these studies were not strong.

The meta-analyses by Tonquati et al (3) included both prospective and retrospective studies and found an overall effect for all adverse self-reported mental health outcomes combined to be 1.28 (95% CI 1.02–1.62). They found estimates for depressive symptoms, anxiety symptoms and poor general mental health symptoms on 1.33, 1.20 and 1.18 respectively, but with highly overlapping CI. The authors mention that, beside the substantial heterogeneity of estimates, the risk of bias was high due to exposure assessment and attrition. Furthermore, different outcome measures may have contributed to the different effect sizes, and the wide CI also in the overall meta-analyses still suggest too small sample size.

Compared to the previously conducted prospective studies, the sample used in this study is, to our knowledge, the largest used to study the association between night shift work and mental health issues, making the estimates very precise and making it possible to study differential effects and provide a range of supplementary analyses.

It has previously been suggested that the association between shift work and adverse mental health depends on sex (15, 17). We tested for but did not find any interaction effect with sex. We also tested for but did not find any interaction effects with age, SES or working hours/week.

Based on our previous study, we had hypothesized that very long hours (>48 hours a week) might be an important risk factor among shift workers. In the stratified analyses (table 1) we did not find a statistically significant increased risk among those who worked >48 hours a week, but we found increased risk among those working 41–48 hours a week. Thus, the hypothesis that “overtime work (>48 hours/week) increases the risk of mental health problems among shift workers” was not confirmed, but results suggested that moderate overtime work may be associated with increased risk for use of psychotropic drugs among night-shift workers. We do not have an explanation for this. However, a possible reason why we did not find any increased risk among those who worked >48 hours may be that this group comprise a small, selective, and particularly healthy group of workers.

We were not able to replicate the finding of differential effects of night-shift work on the rates of antidepressants versus the rates of anxiolytics, and accordingly, we cannot reject that the differential effects we found in our previous study (20) was a coincidence.

Results from the supplementary analyses yielded support for the statement that a more stable and frequent exposure to night shift work may increase the risk for a prescription for psychotropic drugs. We found a higher estimate for employees regularly working in night shifts, but not among those who worked occasionally in shifts. To our knowledge these relationships have not previously been found.

We found no indication that the results were biased by employees treated for former mental health problems, and we found no indication that working in night shifts should increase the risk of relapse for employees with previous use of psychiatric drugs. Neither of these findings have previously been shown.

Results suggested slightly different effects among different industrial groups, with a weaker association among employees in human health and social work activities, and stronger associations among employees within construction work or within the group of ‘other’ industries. Results were, however, not statistically significant for any of the groups and should be replicated in future studies to gain support.

The results from this study should be considered within the national context of shift work in Denmark, where the working environment including shift work is relatively well regulated compared to many other parts of the international labor market. There is in general a high level of influence on decisions important for own work in Denmark (29) and this may have protective features against negative effects of shiftwork. It has been shown that increased influence over one’s own schedule for employees in shiftwork can reduce the adverse consequences on health (30), work–life balance and social life (31).

### Strengths and weaknesses

The study population was followed in national registers covering the entire target population, and accordingly had minimal bias from missing follow up. The problem with reversed causality was minimized through the prospective design and the exclusion of prevalent cases.

Another advantage of the present study is that the number of participants was large enough to (i) focus on shiftwork including night and differentiate between regular and occasional night-shift work (ii) supplement the analysis of psychotropic drug usage with estimated RR for psychiatric hospital treatment and (iii) supplement the analysis of incident use of psychotropic drugs with an analysis of relapse rates among employees with a past record of psychiatric treatment (iv) explore potential interaction effects and provide stratified analyses for gender, age, SES and working hours.

Within-study selection bias was eliminated through our study protocol (23), in which hypotheses and statistical models were specified, peer reviewed, and published before the questionnaire data were linked to the registers.

As suggested by results from previous research, selection processes into (32) and out of shift work (33) are well known, and it is likely that perception of mental health and sleep quality will play a role in both selection processes. Thus, employees experiencing sleeping problems may be less likely to take a job including night shift work, anticipating that it will cause increased sleeping problems. Some employees with mental health issues may likewise avoid night shift work because they fear negative effects, while others (eg, with social phobia) may prefer night shift work. At the same time, employees experiencing increasing mental health problems or sleeping problems while they are working in shift may be less likely to continue working in shifts. These selection processes are likely to have influenced our results in direction of lower estimates of effects than would have been the case if these voluntary selection processes had not taken place.

A drawback of the study is that the response rates have decreased from 70% in 2002 to 53% during the time of inclusion. There were, however, no bias from missing follow-up data, since the endpoints of the study were ascertained in national registers, which covers all residents of Denmark. Previous studies have shown that the response rates to public health questionnaires in Denmark are lower among young men, unmarried people, people with a low educational level and people with an ethnic background other than Danish (35, 36). It is possible that the response rates as well as the reasons for nonresponse in the present study differ between the exposed and unexposed workers.

Furthermore, there may be relevant confounders that were not included in the study. For example, household composition and marital status (37)

Another drawback is, that there may potentially have been classification errors of shift versus day workers in the main analysis. Night-shift workers may have stopped working in night shifts after inclusion and have still been included as a night-shift worker in the analysis until follow-up. And day workers may have started to work night shifts after inclusion but are still included as day workers in the analysis. This drawback may have weakened the contrast between the group of exposed and not exposed workers, and potentially decreased the effect. As results from the first sensitivity analyses showed, the effect was slightly higher for the group who had night-shift work both at the first and the last of their baseline interview rounds, and it was higher for the group with regular night shift work than among those with occasional night-shift work, thus suggesting that a more stable or a more frequent exposure for night shift work may increase the risk. Furthermore, the comparability of findings from this study to our previous study of shift work and psychotropic drug usage has been diminished because we did not use the same categories of shift work as we did in our previous study but analyzed “schedules that include night-shift work” versus “other work schedules (including non-night-shift work and evening work)”.

For further methodological considerations see Hannerz et al (22, 23).

### Concluding remarks

Results from this large prospective study showed a slightly increased incidence for overall psychotropic medicine use among night-shift workers in Denmark, and a slightly higher incidence among night-shift workers with long hours (41–48 hours/week). Although the estimated association in the main analysis was statistically significant, it was still weak (RR <1.2), which implies that night-shift work is not an important predictor of mental ill health in the general working population in Denmark. We found no indication of differential effects for different drugs, but we found some support for higher risk for psychotropic medicine use among those with more stable or more frequent exposure for night shift work.

The current study adds to evidence regarding associations between night shift work and psychiatric treatment. The implication for practice is two-fold. On the one hand, the results suggest that, in a Danish public health perspective, there is no need to be highly concerned about a substantially higher incidence of mental ill health due to night shift work. On the other hand, attention should be paid when mental health issues occur among shift workers and particularly among workers in regular night shift work. Offering opportunities to change from shift to day work for the individual worker may be an important piece of the puzzle to prevent negative mental health issues due to shift work.

The relatively rough categorization of night shift work did not give the opportunity for more detailed analysis of eg, what schedules are associated with less risk. Therefore, future research should use more detailed information on night shift work eg, from payroll data (38).

## Supplementary material

Supplementary material
