# Supplementary material for: Night-shift work and psychiatric treatment. A follow-up study among employees in Denmark
Source: Scand J Work Environ Health. 2022 Mar 31;48(3):200–9. doi: 10.5271/sjweh.4008 (PMC9523462; doi:10.5271/sjweh.4008)
Supplement: Supplementary material [file SJWEH-48-200-S001.pdf]

Night-shift work and psychiatric treatment. A follow-up study among employees in Denmark[1]

by Karen Albertsen, PhD,[2] Harald Hannerz, FilLic, Martin L Nielsen, PhD, Anne Helene Garde, PhD

1. Supplementary material
2. Correspondence to: Karen Albertsen, TeamArbejdsliv ApS, Høffdingsvej 22, 1th, 2500 Valby, Denmark. [E-mail: kal@teamarbejdsliv.dk]

The sensitivity analyses of the present study were conducted in accordance with a statistical analysis plan that was published before we merged the exposure data of the study to its outcome data (1). In the present appendix, we give the results of these analyses together with short descriptions of their rationales and methods. The descriptions of the rationales and methods of the analyses have been adapted (copied and/or slightly amended) from the text in our study protocol, which contains the following copyright and license information:

"©Harald Hannerz, Karen Albertsen, Martin Lindhardt Nielsen, Anne Helene Garde. Originally published in JMIR Research Protocols (http://www.researchprotocols.org), 07.06.2020. This is an open-access article distributed under the terms of the Creative Commons Attribution License (https://creativecommons.org/licenses/by/4.0/), which permits unrestricted use, distribution, and reproduction in any medium, provided the original work, first published in JMIR Research Protocols, is properly cited. The complete bibliographic information, a link to the original publication on http://www.researchprotocols.org, as well as this copyright and license information must be included."

Stable exposure to night-shift work

To find out if the estimated strength of the association between night-shift work and redeemed prescriptions for psychotropic drugs increases when the supposedly harmful exposure to night-shift work is more stable over time, we conducted a sensitivity analysis, which only includes people who (i) participated

in more than one interview, (ii) were between 20 and 59 years old during their last interview, (iii) were employed 32 or more working hours a week according to their first as well as their last interview, and (iv) belonged to the same category in relation to night-shift work (yes versus no) during their last interview as they did during their first interview. The follow-up of the included participants commenced at the very end of the calendar year of their last interview. The statistical model was otherwise the same as in the primary analysis. The results are given in Table S1.

Table S1. Rate ratio (RR) with 99% confidence interval (CI) for incident use of psychotropic drugs, as a function of night-shift work among employees in Denmark in the calendar years 2004 – 2013

Night-shift work	Persons	Person years	Cases	RR*	99% CI
Yes	6659	24 354	762	1.13	1.02 - 1.25
No	77 964	285 037	8237	1.00	-

* Adjusted for sex, age, weekly working hours, calendar time of the interview and socioeconomic status

Occasional versus regular night-shift work

We wanted to know if the estimated strength of association between night-shift work and redeemed prescriptions for psychotropic drugs is greater among participants with regular night-shift work than it is among participants with occasional night-shift work. We therefore conducted a sensitivity analysis where we divided night-shift work into three categories ("no"; "yes, occasionally"; "yes, regularly") and thereafter estimated the rate ratios for the contrasts "yes, occasionally" vs “No” and "yes, regularly" versus "No". The statistical model and inclusion criteria were otherwise the same as in the primary analysis. The results are given in Table S2.

Table S2. Rate ratio (RR) with 99% confidence interval (CI) for incident use of psychotropic drugs, as a function of night-shift work among employees in Denmark in the calendar years 2000 – 2013.

Night-shift work	Persons	Person years	Cases	RR*	99% CI
Yes, regularly	7949	31 872	1071	1.14	1.05 - 1.24
Yes, occasionally	8702	33 685	955	1.03	0.94 - 1.12
No	114 670	456 418	13 800	1.00	-

* Adjusted for sex, age, weekly working hours, calendar time of the interview and socioeconomic status

Inclusion of workers with 28 - 31 working hours a week

In the primary analysis we only included employees who usually worked 32 or more hours a week. There are, however, relatively large groups of night-shift workers in nursing homes, home care etc., whose standard full-time work schedules (e.g. 7 night-shifts, 7 days off-duty) imply an average of only 28 working hours a week. We wanted to know if the estimated effect of night-shift work on the rates of new cases of psychotropic drug use would change if our reference group was changed from 32 - 40 hours a week to 28 - 40 hours a week. We therefore conducted a sensitivity analysis with a redefined inclusion criterion at >= 28 hours a week and a redefined reference group at 28 - 40 hours a week. The statistical model was otherwise be the same as in the primary analysis. The results are given in Table S3.

Table S3. Rate ratio (RR) with 99% confidence interval (CI) for incident use of psychotropic drugs, as a function of night-shift work among employees in Denmark in the calendar years 2000 – 2013, with an average of 28 or more working hours per week

Night-shift work	Persons	Person years	Cases	RR*	99% CI
Yes	17 873	70 268	2218	1.08	1.02 - 1.15
No	124 138	493 275	15 342	1.00	-

* Adjusted for sex, age, weekly working hours, calendar time of the interview and socioeconomic status

Controlling for possible bias due to preexisting mental health problems

In the primary analysis, we excluded participants who received psychiatric treatment (psychiatric hospital treatment or prescriptions for psychotropic drugs) during the calendar year preceding the start of the follow-up period. It was, however, possible that the results of the primary analysis would be influenced by mental health problems that occurred earlier than one year prior to baseline. To explore this possibility, we conducted a sensitivity analysis in which a cohort that excludes all participants who received psychiatric treatment within a one-year period prior to the start of follow-up was stratified into two subcohorts. The first subcohort excludes all participants who received psychiatric treatment within a five-year period prior to the start of follow-up. The second subcohort consists of the participants who received psychiatric treatment within the second to fifth year prior to the start of follow up, but not within the first year. This particular analysis included only participants who lived in Denmark throughout the five-year sampling

period. Moreover, it included only people who participated in Danish Labor Force Survey sometime during the calendar period 2004 – 2013. The statistical methods and inclusion criteria of the analysis were otherwise the same as in the primary analysis. The results are shown in Table S4. The results of the first subcohort (population 2) are interpreted as incidence rate ratios while the results of the second subcohort (population 3) are interpreted as relapse rate ratios.

Table S4. Rate ratio with 99% confidence interval (CI) for incident or recurrent use of psychotropic drugs, as a function of night-shift work among employees in Denmark 2004 – 2013

Type of population*	Night-shift work at baseline	Person years	Cases	RR**	99% CI
1. Workers with no occurrences*** during the first year prior to baseline.	Yes	42 210	1232	1.07	0.99 - 1.16
	No	307 927	8917	1.00	-
2. Workers with no occurrences during the first to fifth year prior to baseline.	Yes	38 953	919	1.08	0.99 - 1.19
	No	285 375	6572	1.00	-
3. Workers with no occurrences during the first year prior to baseline, but at least one occurrence during the second to fifth year prior to baseline.	Yes	3257	313	0.97	0.83 - 1.13
	No	22 553	2345	1.00	-

*Population 2 and 3 are disjoint and exhaustive subsets of population 1.
** Adjusted for sex, age, weekly working hours, calendar time of the interview and socioeconomic status
*** With "occurrences" we mean, "occurrences of redeemed prescriptions for psychotropic medicine or psychiatric hospital treatment".

Controlling for industrial sector

In order to pool results of the present study with results obtained in our previous study (2) we used the same covariates in the primary analysis of the present study as we did in our previous study. The primary analysis therefore controls for an occupational-based SES, but it does not control for industrial sector, which has been shown to be a predictor for mood disorders in the general working population of Denmark (3). We wanted to know if the results of the present study would change if we added industrial sector to the model and conducted therefore a sensitivity analysis where we firstly controlled for (Table S5) and thereafter stratified by (Table S6) industrial sector. The statistical methods and inclusion criteria of the analysis were otherwise the same as in the primary analysis.

Table S5. Rate ratio (RR) with 99% confidence interval (CI) for incident use of psychotropic drugs, as a function of night-shift work among employees in Denmark in the calendar years 2000 – 2013

Night-shift work	Persons	Person years	Cases	RR*	99% CI
Yes	16 651	65 557	2026	1.06	0.99 - 1.13
No	114 670	456 418	13 800	1.00	-

* Adjusted for sex, age, weekly working hours, calendar time of the interview, socioeconomic status and industry

Table S6. Industry specific rate ratio (RR) with 99% confidence interval (CI) for incident use of psychotropic drugs, as a function of night-shift work among employees in Denmark in the calendar years 2000 – 2013

Industry	Night work at baseline	Persons	Person years	Cases	RR*	99% CI
Agriculture, forestry, hunting and fishing	Yes	299	1169	29	1.11	0.66 - 1.86
	No	1813	7486	184	1.00	-
Manufacturing, mining and quarrying	Yes	3222	13 142	388	1.07	0.93 - 1.23
	No	19 558	80 311	2349	1.00	-
Construction	Yes	486	1941	52	1.13	0.78 - 1.64
	No	8343	34 359	820	1.00	-
Wholesale and retail trade; repair of motor vehicles	Yes	976	3898	104	1.09	0.84 - 1.41
	No	15 967	63 836	1708	1.00	-
Transporting and storage	Yes	2250	9063	266	1.07	0.88 - 1.29
	No	5492	22 513	653	1.00	-
Accommodation and food service activities	Yes	411	1588	46	0.96	0.63 - 1.46
	No	1636	6266	212	1.00	-
Human health and social work activities	Yes	3927	14 947	543	1.01	0.89 - 1.14
	No	18 558	72 126	2791	1.00	-
Other	Yes	4823	18 890	565	1.12	1.00 - 1.26
	No	41 814	164 021	4796	1.00	-
Missing	Yes	257	919	33	0.73	0.46 - 1.18
	No	1489	5502	287	1.00	-

* Adjusted for sex, age, weekly working hours, calendar time of the interview and socioeconomic status

Estimated rate ratios without exclusion of prevalent cases

The estimated rate ratios of redeemed prescriptions for psychotropic drugs as a function of night-shift work, without exclusion of prevalent cases are given in Table S7.

Table S7. Rate ratio (RR) with 99% confidence interval (CI) for incident use of psychotropic drugs, as a function of night-shift work among employees in Denmark in the calendar years 2000 – 2013, WITHOUT EXCLUSION OF PREVALENT CASES

Night-shift work	Persons	Person years	Cases	RR*	99% CI
Yes	18 043	67 414	3123	1.08	1.02 - 1.13
No	124 812	469 026	21 877	1.00	-

* Adjusted for sex, age, weekly working hours, calendar time of the interview and socioeconomic status

References

1. Hannerz H, Albertsen K, Nielsen ML, Garde AH. Prospective associations between working time arrangements and psychiatric treatment in Denmark: a study protocol. JMIR Res Protoc. 2020.
2. Hannerz H, Albertsen K. Long working hours and use of psychotropic medicine: a follow-up study with register linkage. Scand J Work Environ Health. 2016 Mar;42(2):153-61. doi: 10.5271/sjweh.3550.
3. Hannerz H, Tüchsen F, Pedersen BH, Dyreborg J, Rugulies R, Albertsen K. Work-relatedness of mood disorders in Denmark. Scand J Work Environ Health. 2009;35(4):294-300.
